# Wearable Textile UHF-RFID Sensors: A Systematic Review

**DOI:** 10.3390/ma13153292

**Published:** 2020-07-24

**Authors:** Chengyang Luo, Ignacio Gil, Raúl Fernández-García

**Affiliations:** Department of Electronic Engineering, Universitat Politecnica de Catalunya, 08222 Barcelona, Spain; chengyang.luo@upc.edu (C.L.); ignasi.gil@upc.edu (I.G.)

**Keywords:** textile, ultra-high frequency (UHF), radio frequency identification (RFID), UHF-RFID sensors, scenario-based

## Abstract

Textile radio-frequency identification operating in ultra-high frequency (UHF-RFID) sensors based on different scenarios are becoming attractive with the forthcoming internet of things (IoT) era and aging society. Compared with conventional UHF-RFID sensors, textile UHF-RFID sensors offer the common textile features, light weight, washability and comfort. Due to the short time and low level of development, researches on the integration of textile UHF-RFID techniques and textile sensing techniques are not flourishing. This paper is motivated by this situation to identify the current research status. In this paper, we provide a systematic review of the fundamentals of textile UHF-RFID sensors techniques, materials, the brief history and the state-of-the-art of the scenario-based development through detailed summary and analysis on the achievements from the starting year of 2004 to the present time. Moreover, according to the analysis, we give a proposal of the future prospects in several aspects, including the new materials and manufacturing processes, machine learning technology, scenario-based applications and unavoidable reliability.

## 1. Introduction

Textile radio-frequency identification operating in ultra-high frequency (UHF-RFID) sensors have been getting more attention since the development of the health-caring field and the calling of the internet of things (IOT) applications because of textiles widely used for everyone and the mature embroidery techniques [1,2]. Moreover, the rapid spread of smart phones, smartbands and other smart devices makes the textile UHF-RFID sensors easier to be connected with individual smart devices by means of RFID modules. Although wearable UHF-RFID sensors on flexible substrates have been focused on for many years, substrate textile materials are still at the early stage due to the short development period since 2004 [3]. Early efforts from textile UHF-RFID techniques were focused on feasibility, reliability and fundamental functions of tags, mainly including identification and tracking for garments in the fabrication process [4]. In recent years since 2012, the research targets of textile UHF-RFID techniques changed from the fundamental researches to tackle scenario-based applications such as exercise tracking [5,6], health-care monitoring [7,8,9], concentration detection of solutions [10], strain capacity [11] and associated applications. In order to achieve these aims, substantial efforts have been devoted to the development of textile sensors integrated into textile UHF-RFID tags. However, although several of the textile UHF-RFID sensors are tested in real scenarios, successful translation to the commercial market has not been completely deployed and these research samples still require further large-scale validation and reliability studies, performance evaluation under stress conditions and device regulatory approvals for safety.

In addition, exploring methods for performance improving, application diversification and high reliability is also an adjoin research proposition, which lead most aspects of techniques to upgrade and even fuse. For these goals, many kinds of tentative studies have been done, such as the metal-pasted thread as the important conductive medium, the graphene-based conductive medium [12,13], and special synthetic textile materials as substrates. Certainly, the use of novel materials will push the fabrication techniques to make changes. The metal-pasted thread can be embroidered on most kinds of textile substrates by traditional embroidery machines with fundamental techniques whereas graphene-based conductive ink needs to be printed on textile substrates by a screen printing technique with specific printing processes [14].

For all of the researches on textile UHF-RFID sensors, although significant technical progress is gradually getting mature, a gap between in-lab researches and mature commercial applications still exists. In addition, combining current fabrication techniques [15,16], the integration of textile UHF-RFID tag and sensing components is also an important issue worth exploring, which will have an impact on future applications.

In order to understand the current research state-of-the-art of the textile UHF-RFID sensors and related applications, this article is written as a brief review. Section 2 presents the fundamentals of textile UHF-RFID sensors including UHF-RFID sensor techniques and electro-textile techniques. Section 3 presents the materials and related impacts in terms of the theory and formulas. Section 4 presents the review search strategy and paper selection. Section 5 presents the development of textile UHF-RFID sensors including the brief history of the textile UHF-RFID tags and sensors, the state of Scenario-based textile UHF-RFID sensors and the state of researches on the reliability of textile UHF-RFID sensors. Section 6 presents the future perspective and Section 7 summarizes the discussion and highlights the main conclusions.

## 2. Fundamentals of Textile UHF-RFID Sensor Techniques

From the early 21st century, the standards and theory of RFID have been improving [17] while the cost of RFID integrated circuits decreases gradually. In addition, the RFID operating bandwidth was defined, as shown in Table 1. The RFID tags can be designed for either a low frequency band (120–150 kHz), high frequency band (13.56 MHz), ultra-high frequency band (433 MHz, 865–868 MHz in Europe, 917–922 MHz in China and 902–928 MHz in North America) and microwave band (2.45–5.8 GHz and 3.1–10 GHz) [18]. In these conditions, RFID sensor applications are becoming more and more extensive in many fields such as stuff identification, logistics tracking, health-care monitoring and so on [19]. Especially for the health-care monitoring and diagnosis field, the textile UHF-RFID sensors, which require some considerable features such as noninvasive detection, comfort, convenience and wireless diagnosis, have become a focal research orientation in the scientific community.

### 2.1. UHF-RFID Sensor Techniques

**Figure 1:**
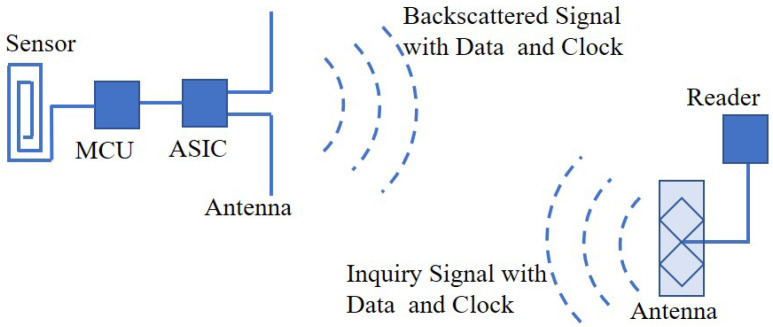
Fundamental of an ultra-high frequency (UHF-RFID) sensor system.

There are many researches on common RFID tags and sensors. The main difference between RFID tag designs for the different operating frequency bands is different structures of RFID antennas. Taking a typical dipole antenna as an example, a classical Formula (1) is as follows,

(1) $$\lambda =\frac{c}{f}$$

where *c* is the speed of light, *f* is the frequency and λ is the wave length. With respect to the formula, the sizes of the dipole antenna are related to the wave length. When the operating frequency is in ultra-high band, the sizes of the RFID antenna need to be adjusted. The fundamental of a UHF-RFID sensor system is shown in Figure 1. The UHF-RFID sensors mainly consist of the UHF antenna, the application specific integrated circuit (ASIC), a micro controller unit (MCU) and some specific sensors. Note that as the essential element in a UHF-RFID sensing system, the UHF antenna has many kinds of structures such as a modified dipole and a folded dipole, which is included in the UHF-RFID sensors tag to receive the inquiry signals including energy [20] from the UHF-RFID reader, and then transmit the measurement data and tag the identification number through radar backscattering mode [21]. Generally, when a continuous data logging is required and the UHF RFID reader cannot supply the power energy, an extra battery needs to be used as an additional power supply to store the measurement data on an additional memory. Once the UHF-RFID reader is presented, the RFID sensor tag downloads all the storage measurement data on the reader.

In order to design a suitable UHF antenna for RFID sensors, the size and impedance need to be considered. Different ASICs have different input impedance. Thus to optimize the energy harvesting efficiency, a perfect conjugate matching between the antenna and the ASICs at operating frequency band is required. The complex impedance of the antenna and the ASICs is shown in Formulas (2) and (3), respectively.

(2) $$Z_{ant}=R_{ant}+jX_{ant}$$

(3) $$Z_{IC}=R_{IC}+jX_{IC}$$

where $Z_{ant}$ and $Z_{IC}$ are the impedances of the antenna and the ASICs, respectively, $R_{ant}$ and $R_{IC}$ are the real parts of the antenna and ASICs, respectively, and $X_{ant}$ and $X_{IC}$ are the imaginary parts of the antenna and ASICs, respectively. When the UHF-RFID sensor is working at a required frequency band, the antenna and the ASICs need to keep conjugate matching as follows,

(4) $$Z_{IC}=Z_{IC}^{*}$$

where $Z_{IC}^{*}$ is the complex conjugate of $Z_{IC}$. Usually the antenna tags are specifically designed taking into account the ASICs input impedance and the RFID tag application environment [22]. As for the whole performance evaluation, some intuitive parameters are considerable, including return loss (S parameters), gain, resonance frequency, bandwidth, radiation power and read range.

### 2.2. Electro-Textile Techniques

**Figure 2:**
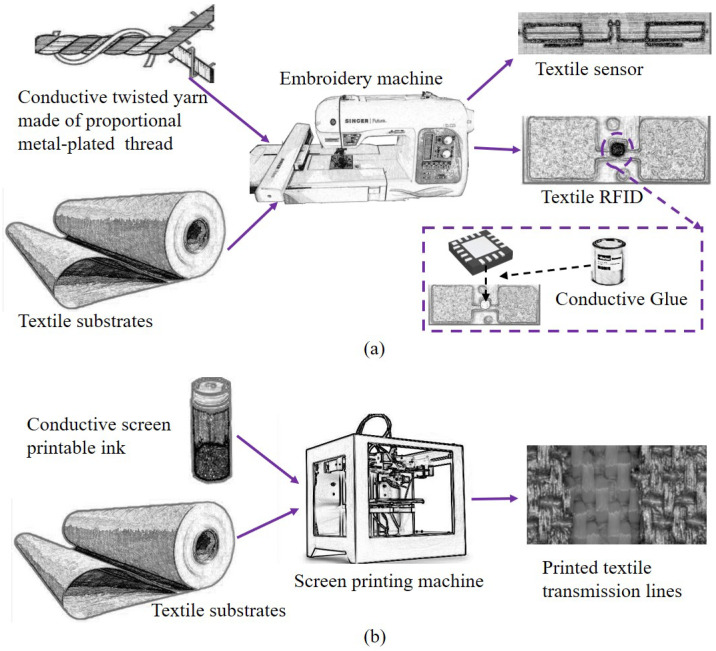
Two typical electro-textile techniques. (**a**) Embroidery technique using conductive yarns, (**b**) screen printing technique using special conductive ink.

The fundamental process of two typical electro-textile techniques is illustrated in Figure 2. As shown in Figure 2a, this technique is worth exploring for the combination of electronics and textiles. The conductive yarns are twisted by a proportional metal-plated thread. By this embroidery technique, the conductive yarns can be embroidered on many kinds of common textile substrates by commercial embroidery machines [23]. Generally, the technological process begins from the model design model simulated by means of an electromagnetic software solver to the model importation into embroidery machines and ending with the manufacturing process. Using this technique for UHF-RFID products, the connection of electronic elements such as ASICs and textile structures can be implemented by commercial glue. There are some studies [24,25,26] on the technique, proving its feasibility for electro-textile RFID or sensors with well ranked performance.

Compared with the embroidery technique using conductive yarns, the screen printing technique using special conductive ink, as shown in Figure 2b, is another popular technique for electro-textile applications but more complex due to its technological process [27]. The essential elements are the specially configured conductive ink and the complex screen printing machines. The general conductive ink is a metal-filled paste with a solid content of a certain percentage, for which the curing process under a required temperature by the screen machine is necessarily performed after the ink is pressed. Although the screen printing technique was invented in ancient China, this technique has been systematically developed in the last 20 years and is employed for the design of robust non-bendable chemical sensors [28], electrochromic materials [29], and non-bendable UHF-RFIDs [30]. However, the invention of conductive compositions for textile printing, described by Ujiie in 2006 [31] that was further developed by Cie in 2015 [32] opens the door for utilization of screen printing in flexible electronics.

## 3. Materials of UHF-RFID Sensors

Compared to common materials such as printed circuit boards (PCB) in UHF-RFID sensor applications, textile materials have quite different physical characteristics, some of which are developed for many novel applications but some pose unavoidable risks. Thus, the investigation of materials of UHF-RFID sensors is necessary for further researches. Especially in recent years, the UHF-RFID sensors based on many textile materials are expected to be applied for human health-caring fields, which need more attention given to safety and reliability.

### 3.1. Materials of Substrates

**Figure 3:**
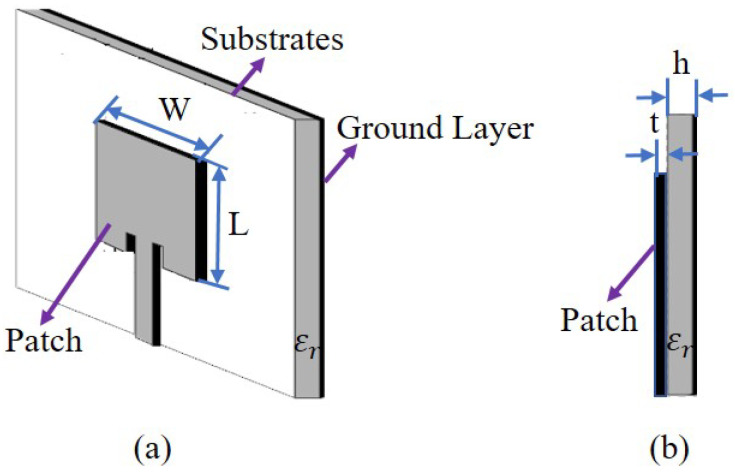
Typical microstrip antenna structure. (**a**) Microstrip antenna. (**b**) Lateral view.

Different materials as substrates of UHF-RFID sensors make variable levels of influence on the design due to different physical characteristics such as dielectric constants, loss tangent, texture, etc. For instance, a typical rectangular microstrip antenna without fringing as shown in Figure 3 can be used as an RFID antenna. With respect to the antenna theory [33,34], the resonant frequency ($f_{r*}$) of the microstrip antenna is a function of its length (L) or width (W). Usually it is given by

(5) $$f_{rL}=\frac{1}{2L\sqrt{\varepsilon_{r}}\sqrt{\mu_{0}\varepsilon_{0}}}=\frac{c}{2L\sqrt{\varepsilon_{r}}}\;\left(L>W\right)$$

or

(6) $$f_{rW}=\frac{1}{2W\sqrt{\varepsilon_{r}}\sqrt{\mu_{0}\varepsilon_{0}}}=\frac{c}{2W\sqrt{\varepsilon_{r}}}\;\left(W>L\right)$$

where $\varepsilon_{r}$ is the dielectric constant of the substrate, $\mu_{0}$ is the permeability of vacuum, $\varepsilon_{0}$ is the permittivity of vacuum and *c* is the speed of light in free space. Therefore, the resonant frequency is also a function of the dielectric constant $\varepsilon_{r}$ of the substrate. When the resonant frequency is set in an ultra-high frequency band, the different materials certainly influence the size of the model designs.

In addition, with regard to loss tangent, some materials with high dielectric constant also have high loss tangent, which increases the energy loss and affects the efficiency of the antenna. As shown in Table 2, some popular materials of UHF-RFID sensors are summarized in terms of the dielectric constant, loss tangent and texture.

**Figure 4:**
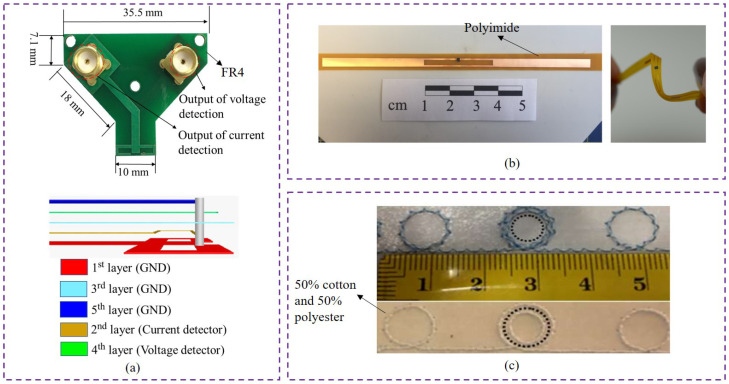
Three typical materials of the substrates. (**a**) Voltage and current sensor with FR4 substrate (adapted from [45]); (**b**) UHF-RFID tag with polyimide substrate (adapted from [40]); (**c**) UHF-RFID tag with 50% cotton and 50% polyester substrate (adapted from [44]).

The texture is also an important feature that determines the types of application orientation and the main parameters for validating reliability. For example, as shown in Figure 4, there are three typical samples for different materials of the substrates. The material of the substrate, as shown in Figure 4a, is FR4, one type of the rigid PCB, which is different with the polyimide, one type of the flexible PCB (FPCB), as shown in Figure 4b, and the 50 % cotton and 50% polyester, one type of the mixed textile, as shown in Figure 4c. Note that the FR4 substrate is so rigid that it cannot be bent easily, meanwhile the polyimide substrate needs to be tested in a bending condition. Compared to the FR4 substrate and polyimide substrate, the textile substrate [44] has to face the challenges of not only the bending impact but also the environmental impacts such as humidity and temperature. However, due to the universality of textile materials in human life, the textile UHF-RFID sensors have a good application foundation.

Certainly, the common materials, PCB, can be applied for multi-sensor structures in terms of multi layers, as shown in Figure 4a, which also gives textile UHF-RFID sensors a novel research orientation towards multi-layer structures with flexible and comfortable features.

### 3.2. Materials of UHF-RFID Antennas and Sensors

As an information and energy transceiver structure, the UHF-RFID antenna is a necessary metallic device for radiating or receiving radio waves in a UHF-RFID sensor. In general, due to the unavoidable lossy nature of the transmission conductors and the reflections losses at several interfaces, there are always conduction-dielectric losses in the real devices. Therefore, the better choice for the conductor is the materials with good conductive ability and the low resistive rate. Currently, metal materials [46], such as copper, aluminum, iron, silver and gold, and synthetic fiber such as graphite fiber [47,48,49] and glass fiber, are popular in the UHF-RFID antennas and sensors designs. Note that metal materials still have better conductive ability than that of some synthetic fiber, thus some synthetic fiber is dealt with metal surface [50] or twisted by a proportional metal-plated fiber [51,52]. Certainly, compared with metal materials, synthetic fiber has the greatest strength of designability. Through advanced modal analysis of structures in specific computer programs, proper structure and performance of synthetic materials can be designed.

In addition, due to the development of printed electronics [53], conductive ink with spacial metal elements such as Nano-Ag [54] and Nano-copper [55] is developed and applied for screen-printed UHF-RFID sensor applications. Currently, conductive inks are key enablers for the use of printing techniques in the fabrication of electronic systems.

## 4. Review Search Strategy and Paper Selection

Full-text articles and conference proceedings were selected from a comprehensive search of PubMed, ScienceDirect, Scopus and IEEE Xplore databases. The search strategy included free text terms and Mesh terms, where suited. These terms were combined using logical Boolean operators. Keywords and their synonyms were combined in each database as follows: (textile OR fabric OR electro-textile) AND (UHF RFID) AND (sensor OR reliability OR feasibility OR sensitivity OR measurement). All results from January 2004 to May 2020 of each database were analyzed and screened to remove duplicates.

The inclusion criteria mainly took into account 5 approaches: (1) The studies that focused on textile UHF-RFID with textile sensing techniques or related reliability researches; (2) the studies that used textile UHF-RFID sensors for certain scenario-based applications; (3) the studies that explored the impact factors on performance for feasibility and reliability of textile UHF-RFID sensors; (4) the papers that are written in English; (5) the selected papers are published in a peer-reviewed journal or presented in a scientific conference.

The exclusion criteria mainly included 2 parts: (1) The papers were not written for reviews or books; (2) the papers only mentioned textile UHF-RFID tags or sensors without reliability researches.

**Figure 5:**
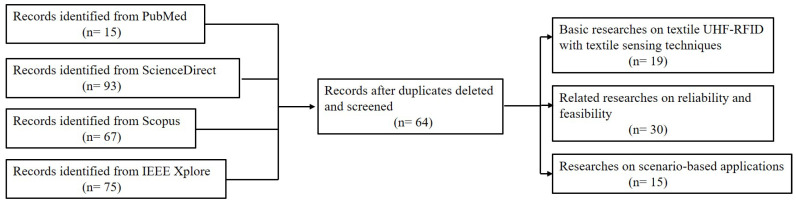
Flow diagram of paper selection.

According to the strategy explained above, the selected papers have been classified, as shown in Figure 5. The literature search returned 250 results and the final total of 64 studies fulfilled the inclusion criteria, of which 29.7% focused on basic researches on textile UHF-RFID with textile sensing techniques, 46.9% were related researches on reliability and feasibility and the remaining 23.4% were exploratory researches on scenario-based applications. From the results, the textile UHF-RFID sensors with more research focused on exploring reliability and feasibility and less on real applications are expected to get more attention and have notable potential in the future.

## 5. Development of Textile UHF-RFID Sensors

Since the standard EPC Gen2 was first published in 2004, the physical and logical requirements for UHF RFID tags and readers have been defined, which give researchers a basic framework to design a UHF-RFID system integrating many kinds of sensors for different applications. In the beginning, in the textile industrial field, the UHF-RFID technique was mainly applied for identification purposes for application such as clothing manufacturing, inventory control, warehousing, distribution, logistics and automatic object tracking. Currently, UHF-RFID tags are gradually used for daily living applications with sensing capabilities.

**Figure 6:**
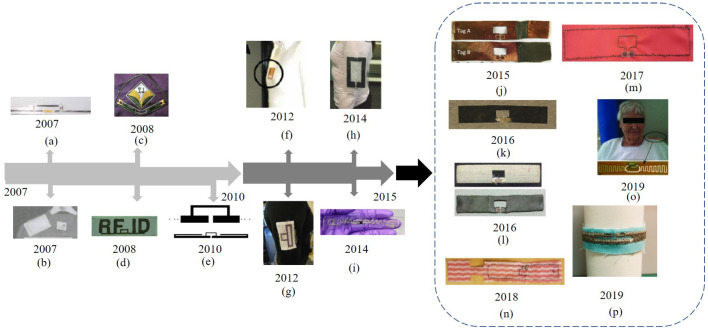
Brief history of textile UHF-RFID and sensing technologies. (**a**) Flexible electro-thread UHF-RFID tag antenna designed using the T-matching method, with a read range of about 2.4 m (adapted from ref. [23]). (**b**) Textile UHF-RFID tag for accessories trace and production process monitor, attached on a garment (adapted from ref. [56]). (**c**) Fabricated ’YJ’ symbol type textile UHF-RFID tag for exploring different shapes of embroidery tag antenna (adapted from ref. [57]). (**d**) Fabricated ’RFID’ symbol textile UHF-RFID tag for analyzing the changes of conductivity for different sewing methods (adapted from ref. [58]). (**e**) Textile UHF-RFID strain sensor for human bodily functions and movement monitoring, fabricated by screen printing the ink on stretchable PVC and on fabric substrates (adapted from ref. [11]). (**f**) Textile UHF-RFID strain sensor for human movement monitoring and feasibility of effective data interaction (adapted from ref. [59]). (**g**) Textile UHF-RFID tag for performance exploration, mainly validated by the read range (adapted from ref. [60]). (**h**) Textile UHF-RFID tag for positioning and localization through recording and analyzing on-body readability and Received Signal Strength (RSS) in an office environment (adapted from ref. [61]). (**i**) E-fiber UHF-RFID broadband tag for tie health information monitor, fabricated on conductive textiles and embedded into polymer (adapted from ref. [62]). (**j**) Textile UHF-RFID strain sensor for exploring the relation between the antenna elongation and its backscatter strength, based on a stretchable antenna made of conductive fabrics (adapted from ref. [63]). (**k**) Graphene-based UHF-RFID tag on a fabric substrate for exploring feasibility and reliability of the low-cost and eco-friendly graphene tag (adapted from ref. [12]). (**l**) Health-care-based UHF-RFID sweat sensor for sweat rate measurements in exercise, by comparing the silver plated sample and the graphene-printed sample (adapted from ref. [6]). (**m**) Health-care-based textile UHF-RFID moisture sensor for body moisture sensing (adapted from ref. [64]). (**n**) Environment-based textile UHF-RFID moisture sensor for humility detection, with a sensor part and an antenna part (adapted from ref. [65]). (**o**) Health-care-based textile UHF-RFID accelerometer sensor for alerting on hospitalized patient bed exits with a super low resolution (adapted from ref. [9]). (**p**) Textile UHF-RFID tag for exploring the impact from geometrical variations and deformations (adapted from ref. [66]).

As shown in Figure 6, a brief history of textile UHF-RFID and sensing technologies preceding current UHF-RFID sensors is provided. From 2007 to 2010, the textile UHF-RFID technique was not yet mature and it was mainly focused on feasibility and simple applications with just tags, such as size-optimizing exploration for the textile UHF-RFID antenna in Figure 6a, accessories trace and production process monitor in Figure 6b and exploration for the relation between conductivity and different sewing methods in Figure 6c,d. Then from 2010 to 2015, the textile UHF-RFID technique was gradually applied for simply sensing uses with just the UHF-RFID antennas and ICs, such as the textile UHF-RFID strain sensors for monitoring human bodily functions and movements, as shown in Figure 6e,f, textile UHF-RFID tag for performance exploration in Figure 6g, RSS-based passive UHF-RFID for indoor localization applications in Figure 6h and textile UHF-RFID with broad impedance bandwidth for tire performance monitoring in Figure 6i. Up to now, textile UHF-RFID integrated with textile sensors has been getting more attention, and many novel textile materials on different kinds of sensing fields have been tested. There are some typical applications, such as UHF-RFID strain sensors with copper-coated fabric, as shown in Figure 6j, graphene-based UHF-RFID sensors for moisture monitoring in Figure 6k and sweat sensing in Figure 6l, silver-plated thread UHF-RFID sensors for environment humidity monitoring in Figure 6m,n, accelerometer-based UHF-RFID sensor for patients’ activity recognition in Figure 6o, and equally important, reliability exploration in Figure 6p.

### 5.1. State-of-the-Art of Textile UHF-RFID Sensors Applications

In modern society, electronic devices are always closely relevant to specific application fields. The rule is also suitable for current researches of textile UHF-RFID sensors. The UHF-RFID technique started to be applied in combination with the textile technique and then textile sensors about 15 years ago. However, up to now there have not been enough research achievements applied for current production and living, which also means the huge development prospect is worth paying attention to.

**Figure 7:**
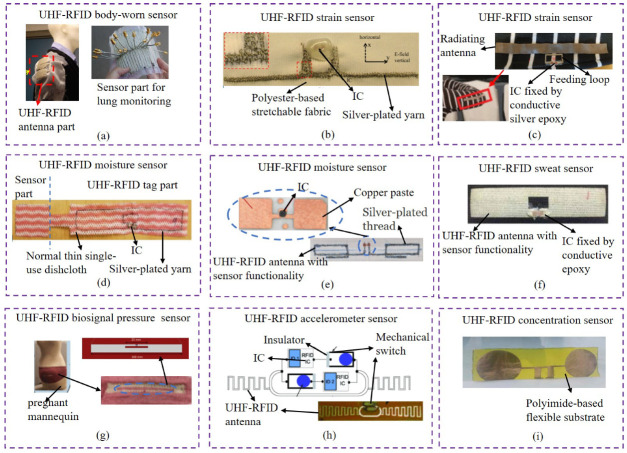
Typical textile UHF-RFID sensor applications. (**a**) Medical-based UHF-RFID body-worn sensor for lung monitoring integrated as part of the garment, with real representation of antenna and sensing array configuration (adapted from ref. [67]); (**b**) deformation-monitoring-based UHF-RFID strain sensor for structural health monitoring applications, with dual-interrogation mode consisting of read range extraction mode and RCS-based sensing mode (adapted from ref. [68] ); (**c**) exercise-based textile UHF-RFID strain sensor for movement monitoring attached on a cotton-based shirt, with a non-stretchable feeding loop and a stretchable radiating antenna (adapted from ref. [69]); (**d**) environment-based textile UHF-RFID moisture sensor for humility detection, with a sensor part and an antenna part (adapted from ref. [65]); (**e**) environment-based textile UHF-RFID moisture sensor for environment humility detection, with a humility sensitive UHF-RFID antenna (adapted from ref. [70]); (**f**) health-care-based textile UHF-RFID sweat sensor for sweat rate measurements, with a tag as the sensing part made by screen printing technique (adapted from ref. [6]); (**g**) health-care-based textile UHF-RFID biosignal pressure sensors for infant heart monitoring, integrated with a modular software framework for interrogation, data storage and post-processing [7,8]); (**h**) health-care-based textile UHF-RFID accelerometer sensor for alerting on hospitalized patient bed exits with a superlow resolution (adapted from ref. [9]); (**i**) textile UHF-RFID concentration sensor for concentration detection using a sensing antenna (adapted from ref. [10]).

A medical-based UHF-RFID body-worn sensor was fabricated and tested for monitoring fluid accumulation in the lungs, which was integrated as part of the garment on various locations such as front, back and shoulders, as shown in Figure 7a [67]. In this work, textiles made by e-fabrics (conductive polymer fibers) were evaluated with a microstrip transmission line structure, which demonstrates the e-fiber transmission line surface had electrical equivalence to metallic but inflexible surfaces of copper transmission lines. Note that some useful fabrication methods were adopted, such as bundled fibers for improving conductivity and assistant yarn for avoiding abrasion damage of the silver coatings on the e-fiber’s polymer core. The important achievement was to use the same e-fibers to fabricate the medical-based UHF-RFID body-worn sensor for lung monitoring. In addition, textile versions were found to be nearly equivalent to the metal one even after being repetitively flexed, washed, and dried. This work gave a pioneering application for textile sensors for lung monitoring in medical-based fields.

For the flexible feature of textiles, a deformation-monitoring-based UHF-RFID strain sensor was proposed for structural health monitoring applications, as shown in Figure 7b [68]. In this work, a novel dual-interrogation mode was applied for the design of the textile UHF-RFID strain sensor, which provided a large identification coding capacity for the UHF-RFID sensor. The dual-interrogation mode consisted of reading range extraction mode for the threshold-power-required chip-enabled approach and RCS-based (radar cross section) sensing mode for the chipless approach. In fact, the range extraction mode relied on the read range changing with the applied strain, while the RCS-based sensing mode was directly linked to the frequency shift depending on the strain changing. Note that here the strain was related to the electrical length of embroidered UHF-RFID sensor structures. This work proved the feasibility of double modes for the design of textile UHF-RFID strain sensors and it is worth considering in future research, but certainly, some important validation measurements such as bending, environment impacts and washing for the performance and reliability need to be considered.

In another example of the textile UHF-RFID strain sensor, as shown in Figure 7c [69], a notable evaluation for the elongation from an attached object was implemented, compared to the last example in Figure 7b. This textile UHF-RFID strain sensor was based on silver-plated material fabricated by plain knitting and designed into two separate parts, the feeding loop and the radiating antenna. This design makes the radiating antenna part fully stretchable while the IC attached to the feeding loop could be non-stretchable, which avoids the reliability challenges caused by mechanical stresses from clothing-integrated electronics. In this work, this textile UHF-RFID strain sensor was integrated on the shirt, the performance of which was examined on-body by means of backscattered signal power measurements under strain and in unloaded conditions. The results revealed that the strain sensitivity was great and the achievements had the potential for future smart monitoring applications. However, for real applications such as a controller in an embodied game, as mentioned in the paper, some safety and reliability validation measurements were expected to be considered, such as the performance impact after washing or working in a high electromagnetic interference (EMI) area.

These above three current kinds of research actually make use of the flexible feature of textile UHF-RFID sensors, which also lead our way to do related researches on this area. In addition to this feature, it is worth knowing that there are many potential features that push the researches on textile UHF-RFID sensors forward.

There is another example for textile UHF-RFID sensors that are sensitive to humidity. As shown in Figure 7d [65], it is an environment-based textile UHF-RFID moisture sensor fabricated on a very common substrate thin single-use dishcloth, which consisted of a sensor part and UHF-RFID antenna part. In this work, the performance of the textile UHF-RFID moisture sensor was evaluated by 10 drops of water from wet state to dry state and the evaluation parameter was the read range in office conditions after 5, 10, and 15 min. The result showed the small changes of the read range from 4.7 m in the dry state and 5.2 m in the wet state. From the result, this textile UHF-RFID moisture sensor had certain moisture detection ability, however the changes of the read range were small relative to the humility from 10 drops of water and the impact from impurities in water also needed to be considered. Thus, this kind of application research had the potential to be focused on in the future.

Compared with the textile UHF-RFID moisture sensor mentioned in Figure 7d, another example of textile UHF-RFID moisture sensors is shown in Figure 7e [70], which is only a textile UHF-RFID tag with moisture sensor functionality. In this work, textile UHF-RFID sensors could curve automatically and permanently after being dipped into the water due to the special material, polyvinyl alcohol (PVA). Note that in contrast to the example in Figure 7d, the test parameter in this work was the change in the backscattered power percentage, which could be measured and compared in order to detect and record the presence of moisture. The comparative measurements in this work proved the results reliable, which showed the potential of the textile UHF-RFID sensors to be applied in environment moisture detection.

In the health-care monitoring field, many kinds of common wearable UHF-RFID sensors on flexible substrates, such as the flexible printed circuit board (FPCB), have been proposed and applied for commercial health-care monitoring applications, whereas textile UHF-RFID sensors for this area are still in the early stages and most investigations for health-care application are at the stage of laboratory research. For example, a health-care-based textile UHF-RFID sweat sensor was proposed for sweat rate measurements, as shown in Figure 7f [6]. In this work, the textile UHF-RFID sweat sensor made by screen printing had a noticeable difference in the response of backscattered signal power. The paper explains that the response curves differences are caused by the conductive antenna impedance and material parameter of the textile substrate changing due to the absorbed sweat. This work indicates the high potential of textile UHF-RFID technology in perspiration sensing, but related sweat components were not analyzed, which were attractive and worth considering.

In another example of the health-care-based textile UHF-RFID biosignal pressure sensors, reliable and secure manner for real-time medical data collection was considered by a software framework, which fills the gap between data safety and textile UHF-RFID sensors for health-care monitoring. As shown in Figure 7g [7,8], the textile UHF-RFID biosignal pressure sensor named bellyband sensor for infant heart monitoring in the paper was applied on a pregnant mannequin driven by proprietary software to simulate various behaviors. In addition, a modular software framework was developed to both interrogate sensor devices and to store that streaming data for live and post-processing. In this work, considering the missed tag reads, two impact factors were found. One was the delay caused by periodic frequency hopping and another one was the greater distance between the tag and the reader. These research achievements are helpful for future applications, but more other impact factors need to be tested such as the comfort and electromagnetic safety for pregnant women and infants.

In addition to textile UHF-RFID sweat sensors for sweat rate measurements and biosignal pressure sensors for infant heart monitoring, another health-care-based textile UHF-RFID accelerometer sensor, as shown in Figure 7h, was proposed for alerting on hospitalized patient bed exits, which could work under a super low resolution. In this work, the sensing device could capture ultra low resolution acceleration data from patients and be analyzed by deep convolutional neural network (CNN) architectures automatically to get the discriminate features. The advances of this work were to combine the textile UHF-RFID accelerometer sensor with neural network approaches, which make the low resolution kinematic sensor possible. This is a mature and useful textile UHF-RFID accelerometer sensor, though the reliability of some components such as the mechanical switch in this sensor device needs to be tested.

The last typical example, as shown in Figure 7i, is a textile UHF-RFID concentration sensor for concentration detection. In this work, the UHF-RFID concentration sensor was printed on a special textile material, which is polyimide flexible substrate, while the sensing antenna was made of copper. The proposed sample is sensitive to the frequency and the concentration of the NaCl solutions and sucrose solutions. Moreover, from the measurement results in the paper, the author proposed that the sensitivity increases with the increase in the percentages of the NaCl and sucrose in water. Although in the work there is no accurate application mentioned, it can give us a research orientation for using total textile UHF-RFID concentration sensors to detect elements in human body fluids.

### 5.2. State of Researches on Reliability of Textile UHF-RFID Sensors

For each technique and related application from start-ups to mature products, the reliability validation is a crucial and necessary step. Currently, for textile UHF-RFID sensors, related reliability researches are constantly advancing with the development of textile UHF-RFID sensors. The reliability researches mainly focus on the impacts from sensitive features of textile UHF-RFID sensors, such as the washing reliability [71,72,73], corrosion-resisting reliability, strain and bending reliability.

Washing reliability is an inevitable research point, which needs to be considered and tested before applied for real applications. The main damage factors for textile UHF-RFID sensors from washing are the mechanical and the chemical impacts. Some works prove that taken separately, neither mechanical constraints nor chemicals used have a significant impact on a silver-plated-nylon yarn on short terms (<30 washes) but the coupling of aggressive chemicals and the mechanical rubbings inside the machine can have a dramatic impact [74]. Certainly, different textile materials for the designs have different degrees of ability to resist the impacts from washing cycles, while if washing for enough times, devices damages are unavoidable and have a direct impact on the read range [75]. In order to keep the function of devices, protective coating materials are expected to be printed on the devices [76], however some textile glues as a fit conformal coating could not always provide good protection [77]. All in all, washing reliability is an important evaluation factor for a good textile UHF-RFID sensor, the impact of which can be reduced by reducing washing cycles or using better protective coating materials.

Corrosion-resisting reliability is another impact factor worth considering for the textile UHF-RFID sensors that are specially applied for sweat and other solution concentration monitoring. The impacts from chemicals for washing are proved small when there are no mechanical rubbings as explained in the last paragraph but complex and corrosive solution and body fluids are proved to have a certain influence on the resistance of textile materials and radiation efficiency of the UHF-RFID antenna in the work [78]. Especially for textile UHF-RFID sweat sensors, due to the complex elements in sweat, the measured data will be interfered with through continuous detection. Currently, painting with conductive paint [72] and developing machine learning technique are some popular methods to reduce this kind of impact.

Strain and bending reliability is also an important factor on performance degradation of textile UHF-RFID sensors although some textile UHF-RFID strain sensors utilize this feature to achieve some health-care monitoring applications [7,8]. However, even for this kind of textile UHF-RFID sensor, the most vulnerable part is the connection of antenna and IC, which can cause total failure of systems when it gets damaged. The sewed and glued interconnections still show strain reliability issues that need to be considered and tested after fabrication [79]. However, when the textile UHF-RFID sensors are applied in harsh conditions, generally a suitable coating would be used to protect the antenna-IC interconnection from mechanical stress. In addition, graphene UHF-RFID on textile substrates is proved to have a remarkable and unique response to high reliability in harsh bending conditions [12], which is another potential choice to solve the problem.

In addition of above three typical reliability problems, many other scenario-based reliability researches need to be explored combining actual situations and applications. Textile UHF-RFID sensors are expected to develop and grow more with the coming of the IoT society and increasing of health-care concerns, meanwhile related safety and reliability of the devices always stay in the spotlight.

## 6. Future Prospects

Textile UHF-RFID sensors are expected to be deployed more on many kinds of fields such as the garment industry, health-caring service industry, sports equipment industry and so on [80]. Due to short development time, textile UHF-RFID sensors still have a long way to go on improving the performance and the futuristic, promising applications and enhancing reliability. Especially for the calling for varieties of IoT applications and the coming-of-age society, more attention needs to be paid to develop useful textile UHF-RFID sensors and close research gaps between laboratory researches and scenario-based textile UHF-RFID sensors.

In the future, the main research focuses in the laboratory are two aspects, one of which is to develop new designs of textile UHF-RFID tags and sensors with common or novel textile materials and manufacturing process [81], and the other to explore novel application scenarios with certain commercial potential.

New designs with novel textile materials and manufacturing processes are always explored but further research on it is still needed. The novel textile materials such as special conductive yarns integrated with graphene as mentioned in [12] and conductive ink on textile substrates [11], are introduced to improve textile UHF-RFID sensor performance and are expected to reduce their cost. Certainly, if the novel textile materials are applied for new designs, related manufacturing processes are needed such as the advanced screen printing technique especially for conductive ink on textile substrates. Compared with the traditional materials and manufacturing processes, the novel ones are still in development and they will require a notable research orientation.

Machine learning technology for textile UHF-RFID sensors is also a novel research direction [82]. As mentioned in [9], a classical machine learning algorithm is used, which is capable of generating probabilistic models using feature vectors extracted from segments. As a part of machine learning, deep neural networks [83] are useful for feature extraction and classifier building when the textile UHF-RFID sensors are applied in some complex conditions for numerous data analysis. Currently, internet of things (IoT) is a hot topic, in which machine learning technology plays an important role pushing textile UHF-RFID sensor techniques to revolutionize the traditional applications. Textile UHF-RFID sensor techniques integrated with the machine learning technology are expected to have a big application market in the future IoT era.

With regard to the scenario-based applications, textile UHF-RFID sensors have great development potential in many different fields of productions and life. Currently, the main application researches focus on the fundamental functions of textile UHF-RFID sensors such as the ID-sensing, strain sensing [84], humidity sensing, sweat sensing and others. However, advanced functions are not covered. For example, there are exercise-based textile UHF-RFID sweat sensors just for sweat sensing without any elements analyzing, on which further researches are worth implementing. Moreover, textile UHF-RFID sensors are more suitable for medical-based applications due to the various medical textile used for patients or the elderly. Many kinds of medical parameters or body fluids can be detected and analyzed by complete textile UHF-RFID sensing systems. The abundant scenarios can create numerous chances for designs and applications of novel textile UHF-RFID sensors in the future.

In addition, reliability is an unavoidable but crucial research direction especially for this kind of flexible and washing-needed component [85]. For example, after fabricating the designs on textile substrates, the performance of UHF-RFID antenna and sensors may decrease such as the resonant frequency shift and gain penalty due to conformal bending or on-body touching. Moreover, environmental factors such as the well-studied humidity and temperature and human factors such as washing and sweat corrosion can imply certain impacts on performance. In the future, the concomitant reliability researches still need to be considered.

## 7. Discussion and Conclusions

Textile UHF-RFID sensors have a very promising prospective for the future society in which many scenario-based applications are needed due to the development of IoT and requirements from aging populations. This review mainly presents the fundamentals and current research state of the textile UHF-RFID sensors and proposes related studies over the lack of further investigation.

According to this review, it can be found that there are many aspects of scenarios, in which current textile UHF-RFID sensors need to be further developed in order to achieve necessary functions with high enough reliability. Apart from fundamental functions that are based on the basic features of textile UHF-RFID tags and sensors, further researches on more complex application such as monitoring and diagnosis of medical parameters should be addressed. In order to achieve these targets, novel materials with advanced manufacturing processes are worth exploring and cross-domain integration with machine learning creates a novel research direction.

In conclusion, the textile UHF-RFID sensor technique as a branch of traditional UHF-RFID sensor techniques offers special advantages such as common use of textile materials in many areas and it is expected to continuously attract research efforts based on different scenarios in the forthcoming IoT era and the aging society.

## Figures and Tables

**Table 1:** RFID operating bands.

Band	Range	Regulations	Typical Use
120–150 kHz (LF)	10 cm	Unregulated	Animal identification, factory data collection
13.56 MHz (HF)	10 cm–1 m	ISM band worldwide	Smart cards
433 MHz (UHF)	1–100 m	Short range devices	Defense applications
433 MHz (UHF)	1–100 m	Short range devices	Defense applications
865–868 MHz (Europe) and 902–928 MHz (North America) (UHF)	1–12 m	ISM band	staff identification, logistic tracking
2.45–5.8 GHz (microwave band)	1–2 m	ISM band	802.11 WLAN, Bluetooth standards
3.1–10 GHz (microwave band)	Up to 200 m	Ultra wide band	Active tags

**Table 2:** Materials of substrates.

Material	Type	Dielectric Constant	Loss Tangent	Texture	Example
FR4	PCB	4.4	0.02	Rigid	[35,36]
Rogers	PCB	2.2	0.0009	Rigid	[37,38]
Polyimide	FPCB	3.5	0.0027	Flexible	[39,40]
Biodegradable paper	FPCB	3.2	0.05	Flexible	[41]
Polyester	textile	3.2	0.003	Flexible	[42,43]
